# Severe maternal morbidity in pregnancies complicated by maternal seizure disorder

**DOI:** 10.1002/pmf2.70466

**Published:** 2026-09-29

**Authors:** Janki Amin, Brianna Lau, Brandie D. Taylor, Calla Holmgren, Rachel K. Harrison

**Affiliations:** ^1^ Department of Obstetrics and Gynecology Advocate Aurora Health Chicago Illinois USA; ^2^ Chicago Medical School Rosalind Franklin University of Medicine and Science Chicago Illinois USA; ^3^ Department of Obstetrics and Gynecology University of Texas Medical Branch Galveston Texas USA; ^4^ Department of Maternal Fetal Medicine Advocate Aurora Health Chicago Illinois USA

**Keywords:** hypertensive disorders of pregnancy, maternal death, pregnancy, seizure disorder, severe maternal morbidity

## Abstract

**Introduction:**

Seizure disorders are a common comorbidity of pregnancy, leading to increased maternal and neonatal complications. In particular, we sought to identify the impact of seizure disorders on severe maternal morbidity (SMM) and hypertensive disorders of pregnancy in a contemporary cohort.

**Methods:**

Retrospective cohort study using the National Inpatient Sample (NIS) database from 2017 to 2021 through Healthcare Cost and Utilization Project (HCUP)/Agency for Healthcare and Research Quality (AHRQ), utilizing delivery admissions. Pregnant patients with seizure disorder were identified by International Classification of Diseases (ICD)‐10 codes. We assessed the prevalence of seizure disorders in pregnancy and compared baseline characteristics of those with a seizure disorder to those without a seizure disorder using weighted sampling per the NIS guidelines. Our primary outcome was a composite of SMM as defined by the Centers for Disease Control. Secondary outcomes were hypertensive disorders of pregnancy, hemorrhage, cesarean delivery, maternal death, and maternal length of stay. Statistical analyses were performed with survey weights and included Student's *t*‐test, *χ* ^2^ analysis, and linear and logistic regression to control for confounders. A sensitivity analysis was performed among only subjects with HDP to assess adverse outcomes in this population.

**Results:**

Over a 5‐year period, more than 16 million subjects were included, of which a total of 83,050 (0.5%) pregnant patients had an ICD‐10 diagnosis for seizure disorder at the time of delivery. The prevalence increased from 2017 to 2021 (0.45% to 0.54%, *p* < 0.001). Patients with an ICD‐10 code for seizure disorder were more likely to use tobacco and have ICD‐10 or CMR code for obesity, diabetes mellitus, and hypertension. Those with a seizure disorder were more likely to experience the composite SMM, hemolysis, elevated liver enzymes, low platelets (HELLP) syndrome, eclampsia, hemorrhage, cesarean delivery, maternal death, and longer length of stay than those without a seizure disorder during their delivery hospitalization. In the adjusted analyses, seizure disorder was associated with SMM (adjusted odds ratio [aOR], 2.16; 95% confidence interval [CI], 2.03–2.30) and maternal death (aOR, 6.30; 95% CI, 3.07–12.942). Having a seizure disorder was also associated with hemorrhage, cesarean, severe forms of hypertensive disorders of pregnancy, and increased length of stay. SMM remained elevated in the seizure disorder population in the sensitivity analysis.

**Conclusion:**

Seizure disorder in pregnancy increased in prevalence from 2017 to 2021 and is associated with risk factors such as obesity, tobacco use, diabetes mellitus, and hypertension. These patients are more likely to experience SMM, death, and severe forms of hypertensive disorders of pregnancy than pregnant patients without a seizure disorder.

## 1 INTRODUCTION

Seizure disorder affects 1.5 million people of reproductive potential in the United States with an estimated 24,000 affected people giving birth each year [1]. While studies have demonstrated no significant reduction in fertility among patients with pre‐existing seizure disorders, interactions between anti‐seizure medications and oral contraceptives may reduce contraceptive efficacy and contribute to an increased risk of unintended pregnancy in this population. Indeed, more than 50% of pregnancies among patients with seizure disorders have been reported to be unintended [2]. Despite these considerations, the majority of patients with seizure disorders—more than 90%—experience no pregnancy‐related complications [3].

Between 14% and 32% of pregnant people with seizure disorder experience an increase in seizure frequency during pregnancy compared to their prepregnancy baseline; guidelines from both the American College of Obstetricians and Gynecologists (ACOG) [4] and the American Academy of Neurology (AAN) [5] acknowledge the elevated pregnancy risks in this patient population. However, guidelines remain limited regarding the magnitude and nature of maternal complications and antepartum management recommendations. A 2009 structured literature review by a 20‐member committee of neurologists, epileptologists, and pharmacologists using the AAN prognostic classification of evidence analyzed 285 clinical studies of pregnant people with seizure disorders taking anti‐seizure medications and found no substantial increased risk of cesarean delivery, late‐pregnancy bleeding, or preterm labor compared with patients without seizure disorders [6]. In contrast, a 2015 retrospective study of the 2007–2011 National Inpatient Sample (NIS) database that included 69,385 people with seizure disorders (regardless of anti‐seizure medication usage) and 20,449,532 people without seizure disorder reported an increased risk of maternal mortality, severe maternal morbidity (SMM), higher rates of cesarean section, and increased hospital length of stay [7]. A 2022 longitudinal study that included 2,748,784 participants found that those with a seizure disorder who received anti‐seizure medications during pregnancy were not at increased risk for mortality, but the risk of SMM in this group, defined by criteria from the Centers for Disease Control and Prevention (CDC) [8], was approximately double compared to patients without seizures [9]. This study population was based on insurance and pharmacy data within 1 year prior to delivery hospitalization.

Lastly, hypertensive disorders of pregnancy (HDP) and the associated complications among patients with seizure disorders have been previously examined in large cohorts, but the severity of preeclampsia including rates of HELLP syndrome or preeclampsia with severe features has not been fully elucidated nor the impact of HDP on SMM in this population. Additionally, most of the data are from more than 10 years ago and many of the more recent, larger studies were performed outside of the United States [7, 10, 11].

These variable findings, compounded by differences in outcome definitions and study design, highlight the need for contemporary national data. Therefore, this study aims to (1) characterize the prevalence of seizure disorders in pregnancy within a contemporary national cohort, (2) assess rates of CDC‐defined SMM [8] among pregnant patients with and without seizure disorder, (3) characterize the type and severity of HDP among those with seizure disorder, and (4) assess SMM among those with HDP.

## 2 METHODS

This is a retrospective cohort study utilizing the National Inpatient Sample (NIS), Healthcare Cost and Utilization Project (HCUP), and Agency for Healthcare and Research Quality between 2017 and 2021. The NIS is a large, national database representing all payers and includes 20% stratified sample of inpatient admissions with baseline hospital and patient level data elements as well as International Classification of Diseases (ICD)‐10 diagnosis and procedure codes [12]. Survey weights were applied based on NIS methods for national estimations.

Participants were included if they were between the ages of 11 and 50 years with a pregnancy‐associated ICD‐10 diagnosis code. Only those with a gestational age of 20 weeks or greater and a delivery encounter were included. Subjects with missing baseline data or unknown gestational age were excluded. Groups were then separated based on the presence or absence of ICD‐10 codes for seizure disorder (G40.000‐G40.319 G40.A G40.A00‐G40.A19 G40.B G40.B00‐G40.B19 G40.C G40.C00‐G40.C19 G40.4 G40.400‐G40.509 G40.8 G40.800‐G40.890 G40.9 G40.900‐G40.919). ICD‐10 codes O99.350‐O99.359 were not used as they were considered too vague. Baseline characteristics utilized were either as defined by the NIS database, CMR, or DRG diagnoses within the database, or ICD‐10 codes (Supplement 1). These included maternal age, race/ethnicity, presence of comorbid health conditions, patient location and income, and hospital characteristics including ownership, bed size, teaching status, and year of admission.

The primary outcome was SMM and mortality using ICD‐10 codes as defined by the CDC [8] (Supplement 1 in the Supporting Information). Secondary outcomes included CDC SMM excluding transfusion, CDC SMM excluding eclampsia, gestational hypertension, preeclampsia, abruption, HELLP, and eclampsia, as well as fetal growth restriction, abruption, fetal demise, mode of delivery, hemorrhage, preterm delivery, gestational age at delivery, and death during admission. In this context, the secondary outcomes are considered exploratory due to our evaluation of multiple secondary outcomes. Those with ICD‐10 codes for seizure disorder were compared to those without using weighted sampling with survey statistics for baseline characteristics and outcomes. A generalized linear model and logistic regression were utilized to complete the regression analysis for delivery hospitalization outcomes using unadjusted and adjusted models to generate odds ratios (ORs) and 95% confidence intervals (CIs). A sensitivity analysis, including only those with HDP, was performed to isolate rates of SMM and other outcomes in this higher‐risk population.

Directed acyclic graphs (DAGs) were used to identify a minimally sufficient adjustment set based on subject‐matter knowledge of the relationships among seizure disorder, demographic characteristics, comorbid conditions, residential urbanicity, and SMM, while avoiding adjustment for variables that may lie on the causal pathway between exposure and outcome [13, 14]. Final models were adjusted for age, chronic hypertension, income, insurance status, residential urbanicity, mood disorders, obesity, and race based on the results of the DAG (Supplement 2 in the Supporting Information).

Race and ethnicity are not biological variables; however, race was included as a proxy for exposure to structural racism, whereas insurance status, income, and residential urbanicity were included as proxies for social determinants of health [15]. Although the temporal relationship between seizure disorder and certain chronic health conditions cannot be established in this dataset, obesity and chronic hypertension were retained in the adjustment set because they are common comorbidities among individuals with seizure disorder and are also established risk factors for SMM [16]. Residual confounding remains possible, particularly because direct measures of important social and environmental factors were unavailable. To evaluate the potential impact of unmeasured confounding, E‐values were calculated as a sensitivity analysis [17] (Supplement 3 in the Supporting Information). The E‐value estimates the minimum strength of association that an unmeasured confounder would need to have with both the exposure and outcome, beyond the measured covariates, to fully explain the observed association. All analyses were performed using Stata (Version 17.0, StataCorp LLC).

## 3 RESULTS

Over 16 million pregnant patients were included in our study. Of this sample, 83,050 (0.5%) patients had an ICD‐10 code diagnosis for seizure disorder. Baseline characteristics are provided in Table 1. On average, patients with seizure disorder had a mean age of 28.3 years (95% CI, 28.2–28.4 years) while those without seizure disorder had a mean age of 29.1 years (95% CI, 29.1–29.12 years). Patients with seizure disorder were more likely to be non‐Hispanic White (60.3% vs. 52.5%) or non‐Hispanic Black (18.6% vs. 15.0%) than patients without seizure disorder, but less likely to be Hispanic (15.1% vs. 21.1%) or Asian/Pacific Islander (2.2% vs. 6.2%). Patients with seizure disorder were more likely to have an ICD‐10 code for obesity than their counterparts without seizure disorder (16.4% vs. 12.7%).

**TABLE 1:** Baseline characteristics comparing those with and without a history of seizure disorder.

Total (weighted): *N* = 16,708,857	No history of seizure disorder (*N* = 16,625,807)	History of seizure disorder (*N* = 83,050)	OR (95% CI)
Age	29.1 ± 0.0	28.3 ± 0.0	−0.78 (−0.87 to −0.69)
Race			
Non‐Hispanic White	8,728,549 (52.5)	50,079 (60.3)	Ref
Non‐Hispanic Black	2,493,871 (15.0)	15,447 (18.6)	1.08 (1.04–1.12)
Hispanic	3,508,045 (21.1)	12,541 (15.1)	0.62 (0.60–0.65)
Asian/pacific Island	1,030,800 (6.2)	1827 (2.2)	0.31 (0.27–0.34)
Native American	116,381 (0.7)	747 (0.9)	1.10 (0.93–1.30)
Other	748,161 (4.5)	2408 (2.9)	0.57 (0.52–0.62)
Obesity (BMI 30 kg/m^2^ or higher)	2,111,477 (12.7)	13,620 (16.4)	1.35 (1.29–1.41)
Tobacco use	814,665 (4.9)	10,963 (13.2)	2.96 (2.83–3.09)
Diabetes mellitus	731,536 (4.4)	4900 (5.9)	1.37 (1.29–1.46)
Chronic hypertension	581,903 (3.5)	5730 (6.9)	2.04 (1.92–2.17)
Asthma	947,671 (5.7)	11,959 (14.4)	2.75 (2.63–2.87)
Anemia	2,427,368 (14.6)	15,032 (18.1)	1.30 (1.25–1.35)
Mood disorder	1,396,568 (8.4)	21,012 (25.3)	3.71 (3.58–3.84)
History of cesarean	2,976,019 (17.9)	16,527 (19.9)	1.14 (1.10–1.18)
Patient location by population			
Central metro > 1million	5,553,020 (33.4)	24,417 (29.4)	Ref
Fringe metro > 1million	4,123,200 (24.8)	19,018 (22.9)	1.05 (1.01–1.09)
Population 250k to 999k	3,408,290 (20.5)	18,437 (22.2)	1.23 (1.18–1.28)
Population 40k to 250k	1,413,194 (8.5)	8222 (9.9)	1.32 (1.25–1.39)
Micropolitan	133,006 (0.8)	7640 (9.2)	1.36 (1.28–1.44)
Not metro or micropolitan	847,916 (5.1)	5315 (6.4)	1.42 (1.33–1.52)
Household income quartile (by zip code)			
First quartile (bottom)	4,605,349 (27.7)	28,984 (34.9)	1.76 (1.68–1.84)
Second quartile	4,189,703 (25.2)	22,340 (26.9)	1.49 (1.42–1.56)
Third quartile	4,106,574 (24.7)	18,354 (22.1)	1.25 (1.19–1.31)
Fourth quartile (top)	3,724,180 (22.4)	13,371 (16.1)	Ref
Insurance Type			
Medicare	116,381 (0.7)	3322 (4.0)	8.42 (7.76–9.13)
Medicaid	6,999,465 (42.1)	44,432 (53.5)	1.71 (1.66–1.77)
Private	8,678,671 (52.2)	32,140 (38.7)	Ref
Self‐pay	399,019 (2.4)	1163 (1.4)	0.79 (0.69–0.90)
No charge	0 (0.0)	0 (0.0)	0.55 (0.23–1.33)
Other	432,271 (2.6)	1993 (2.4)	1.25 (1.13–1.38)
Hospital location/ teaching status			
Rural	1,413,194 (8.5)	7724 (9.3)	Ref
Urban non‐teaching	3,075,774 (18.5)	12,541 (15.1)	0.75 (0.70–0.80)
Urban teaching	12,136,839 (73.0)	62,786 (75.6)	0.95 (0.90–1.00)
Hospital type and ownership			
Government	1,928,594 (11.6)	11,378 (13.7)	Ref
Private, non‐profit	12,485,981 (75.1)	62,370 (75.1)	0.85 (0.81–0.89)
Private profit	2,211,232 (13.3)	9302 (11.2)	0.71 (0.67–0.75)
Hospital bed size			
Small	3,291,910) (19.8)	14,949 (18.0)	Ref
Medium	4,971,116 (29.9)	22,673 (27.3)	1.00 (0.96–1.05)
Large	8,362,781 (50.3)	45,428 (54.7)	1.19 (1.14–1.24)
Year			
2017	3,391,665 (20.4)	15,281 (18.4)	Ref
2018	3,391,665 (20.4)	16,610 (20.0)	1.09 (1.04–1.15)
2019	3,358,413 (20.2)	17,025 (20.5)	1.13 (1.07–1.18)
2020	3,242,032 (19.5)	16,693 (20.1)	1.15 (1.09–1.21)
2021	3,242,032 (19.5)	17,441 (21.0)	1.20 (1.14–1.26)

*Note*: Data presented as *N* (%) or mean ± standard error.

Abbreviations: BMI, body mass index; CI, confidence interval; OR, odds ratio.

Patients with a seizure disorder were more likely to have ICD‐10 diagnosis codes for tobacco use, previous cesarean delivery, diabetes mellitus, hypertension, asthma, and mood disorders. They were also more likely to be in the lowest household income quartile and deliver at an urban teaching hospital (Table 1).

With regard to the primary outcome, patients with a history of seizure disorder had a higher rate of the composite of SMM (6.2% vs. 2.6%; OR, 2.49; 95% CI, 2.33–2.65). With regard to the secondary outcomes, patients with a history of seizure disorder had a higher rate of hemorrhage (5.5% vs. 4.7%; OR, 1.18; 95% CI, 1.10–1.26), cesarean delivery (38.2% vs. 32.4%; OR, 1.29; 95% CI, 1.25–1.33), maternal death (5 in 10,000 vs. 0.6 in 10,000; OR, 8.05; 95% CI, 3.97–16.33), and maternal length of stay in the hospital (3.0 days vs. 2.6 days). Rates of HDP were higher among those with seizure disorder compared to those without (17.8% vs. 13.1%, OR, 1.45; 95% CI, 1.39–1.50). In particular, rates of preeclampsia with severe features, eclampsia, and HELLP syndrome were higher (Table 2).

**TABLE 2:** Pregnancy outcomes comparing those with and without a history of seizure disorder.

	No history of seizure disorder (*N* = 16,625,807)	History of seizure disorder (*N* = 83,050)	OR/Coef	95% CI	OR/Coef^*^	95% CI^a^
Gestational age at delivery	38.3 ± 0.0	37.8 ± 0.0	−0.56	−0.59 to −0.51	−0.41	−0.44 to −0.37
Preterm delivery	1,679,207 (10.1)	13,703 (16.5)	1.76	1.69–1.84	1.49	1.43–1.55
Cesarean section delivery	5,386,761 (32.4)	31,725 (38.2)	1.29	1.25–1.33	1.25	1.21–1.29
Length of stay (days)	2.6 ± 0.0	3.0 ± 0.0	0.43	0.39–0.46	0.33	0.30 to 0.36
Hemorrhage	781,413 (4.7)	4568 (5.5)	1.18	1.10–1.26	1.15	1.07–1.23
Suspected FGR	648,406 (3.9)	5232 (6.3)	1.67	1.57–1.78	1.48	1.37–1.56
IUFD	83,129 (0.5)	498 (0.6)	1.32	1.13–1.56	1.07	0.88–1.30
Abruption	182,884 (1.1)	997 (1.2)	1.42	1.26–1.60	1.25	1.11–1.42
Any hypertensive disorder of pregnancy	2,177,981 (13.1)	14,783 (17.8)	1.45	1.39–1.50	1.26	1.21–1.31
Gestational hypertension	1,130,555 (6.8)	5980 (7.2)	1.08	1.02–1.14	0.99	0.93–1.05
Preeclampsia without severe features	249,387 (1.5)	1329 (1.6)	1.12	0.99–1.27	1.07	0.95–1.21
Preeclampsia with severe features	432,271 (2.6)	3239 (3.9)	1.53	1.42–1.66	1.33	1.23–1.44
Preeclampsia unspecified	249,387 (1.5)	1661 (2.0)	1.36	1.22–1.51	1.28	1.14–1.42
Superimposed preeclampsia	182,884 (1.1)	1661 (2.0)	1.83	1.64–2.04	1.09	0.96–1.23
Eclampsia	9975 (0.06)	166 (0.2)	28.63	25.30–32.40	23.76	20.90–27.00
HELLP	49,877 (0.3)	498 (0.6)	2.09	1.70–2.56	1.99	1.62–2.44
SMM	432,271 (2.6)	5149 (6.2)	2.49	2.33–2.65	2.16	2.03–2.30
SMM excluding transfusion	266,013 (1.6)	3903 (4.7)	3.08	2.86–3.31	2.60	2.42–2.80
SMM excluding eclampsia	432,271 (2.6)	4319 (5.2)	2.04	1.90–2.17	1.82	1.70–1.95
Death	6/100,000	50/100,000	8.05	3.97–16.33	6.30	3.07–12.94

*Note*: Data presented as mean ± SE or *N* (%).

Abbreviations: CI, confidence interval; FGR, fetal growth restriction; IUFD, intrauterine fetal demise; HELLP, hemolysis, elevated liver enzymes, low platelets; OR, odds ratio; SMM, severe maternal morbidity.

^a^ Controlled for: age, race/ethnicity, obesity, chronic hypertension, mood disorder, patient location, income quartile, insurance.

In the adjusted regression analyses, patients with seizure disorder had 2.16‐fold increased odds of experiencing any SMM than patients without seizure disorder (95% CI, 2.03–2.30). Patients with seizure disorder were 2.60 times more likely to experience SMM excluding transfusion than patients without seizure disorder (95% CI, 2.42–2.80). The adjusted analysis showed over sixfold increased odds of maternal death in patients with seizure disorder when compared to those without seizure disorder (adjusted odds ratio [aOR], 6.30; 95% CI, 3.07–12.94).

Patients with seizure disorder were also more likely to experience any HDP than patients without seizure disorder (aOR, 1.26; 95% CI, 1.21–1.31). There was no significant difference between groups in the development of gestational hypertension or preeclampsia without severe features; however, patients with seizure disorder had 1.33‐fold increased odds of developing preeclampsia with severe features than patients without seizure disorder (95% CI, 1.23–1.44). They were 23.76‐times more likely to experience eclampsia (95% CI, 20.90–27.00) and 1.99‐times more likely to develop HELLP syndrome than patients without seizure disorder (95% CI, 1.62–2.44) (Table 2, Figure 1).

**FIGURE 1:**
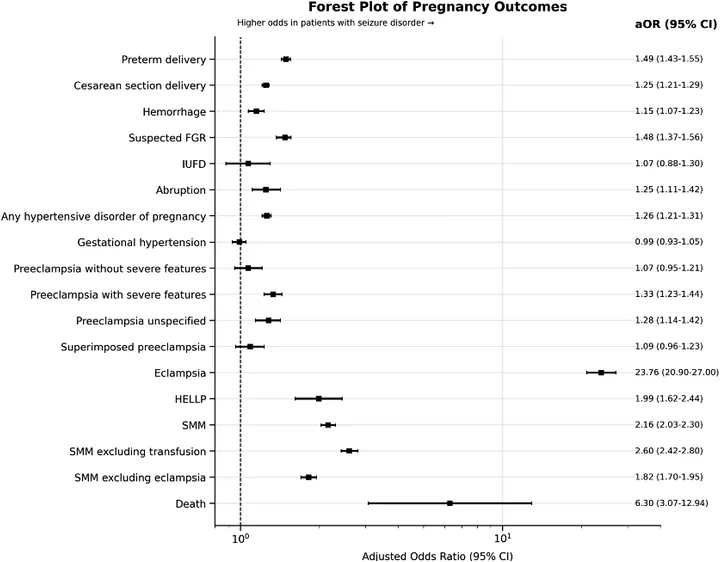
Forest plot of outcomes including all subjects.

Patients with seizure disorder were more likely to deliver at an average earlier gestational age than patients without seizure disorder (38.3 vs. 37.8 weeks; adjusted linear regression coefficient [aLRC], −0.41; 95% CI, −0.44 to −0.37). Of the patients with seizure disorder, there was a 1.49‐times increased likelihood of preterm delivery (16.5% in the seizure disorder group and 10.1% in the non‐seizure disorder group, 95% CI. 1.43–1.55). They were 1.48‐times more likely to have a suspected growth‐restricted fetus than patients without seizure disorder (95% CI, 1.37–1.56) (Table 2, Figure 1).

When looking at delivery and postpartum maternal outcomes, patients with a seizure disorder had a 1.25‐fold increased risk of having a cesarean delivery (95% CI, 1.21–1.29). In the postpartum period, they were more likely to have a postpartum hemorrhage than patients without seizure disorder (aOR, 1.15; 95% CI; 1.07–1.23). On average, patients without a history of seizure disorder stayed in the hospital for 2.6 days after delivery, whereas patients with a history of seizure disorder stayed for 3.0 days (aOR, 0.33; 95% CI, 0.30–0.36).

In the sensitivity analysis incorporating only patients with an ICD‐10 diagnosis code for HDP, many of these findings persisted, including the association of seizure disorder with SMM, SMM excluding transfusion, SMM excluding eclampsia, preterm delivery, cesarean delivery, and increased length of stay (Tables 3, 4, Figure 2).

**TABLE 3:** Baseline characteristics among those with hypertensive disorders of pregnancy only.

Total (weighted): *N* = 2,184,454	No history of seizure disorder (*N* = 2,169,644) act	History of seizure disorder (*N* = 14,810)	OR (95% CI)
Age	29.2 ± 0.0	28.5 ± 0.1	−0.78 (−1.00 to −0.56)
Race			
Non‐Hispanic White	1,149,911 (53.0)	8146 (55.0)	Ref
Non‐Hispanic Black	423,081 (19.5)	3495 (23.6)	1.16 (1.06–1.27)
Hispanic	407,893 (18.8)	2251 (15.2)	0.78 (0.70–0.87)
Asian/pacific Island	88,955 (4.1)	281 (1.9)	0.45 (0.34–0.59)
Native American	17,357 (0.8)	133 (0.9)	1.11 (0.75–1.63)
Other	82,446 (3.8)	504 (3.4)	0.87 (0.71–1.06
Obesity (BMI 30 kg/m^2^ or higher)	527,223 (24.3)	3851 (26.0)	1.10 (1.01–1.19)
Chronic hypertension	149,705 (6.9)	1614 (10.9)	1.66 (1.48–1.87)
Diabetes mellitus	212,625 (9.8)	1570 (10.6)	1.10 (0.97–1.23)
Tobacco use	110,652 (5.1)	1614 (10.9)	2.26 (2.01–2.54)
Asthma	171,402 (7.9)	2147 (14.5)	1.97 (1.78–2.18)
Anemia	(384,027 (17.7)	3169 (21.4)	1.27 (1.16–1.39)
Mood disorder	258,188 (11.9)	4058 (27.4)	2.79 (2.57–3.02)
History of cesarean	342,804 (15.8)	2592 (17.5)	1.13 (1.02–1.24)
Type of HDP			
Gestational hypertension	1,123,876 (51.8)	6013 (40.6)	0.64 (0.59–0.69)
Preeclampsia without severe features	243,000 (11.2)	1363 (9.2)	0.80 (0.71–0.91)
Preeclampsia with severe features	433,929 (20.0)	3273 (22.1)	1.13 (1.04–1.24)
Preeclampsia unspecified	249,509 (11.5)	1674 (11.3)	0.99 (0.88–1.11)
Superimposed preeclampsia	180,080 (8.3)	1629 (11.0)	1.36 (1.22–1.53)
Eclampsia	10,848 (0.5)	1451 (9.8)	22.73 (19.99–25.85)
HELLP	45,563 (2.1)	474 (3.2)	1.54 (1.25–1.89)
Patient location by population			
Central metro > 1million	742,018 (34.2)	4872 (32.9)	Ref
Fringe metro > 1million	525,054 (24.2)	3288 (22.2)	0.95 (0.86–1.05)
Population 250k to 999k	451,286 (20.8)	3199 (21.6)	1.08 (0.97–1.19)
Population 40k to 250k	177,911 (8.2)	1437 (9.7)	1.23 (1.08–1.40)
Micropolitan	162,723 (7.5)	1200 (8.1)	1.13 (0.98–1.30)
Not metro or micropolitan	110,652 (5.1)	815 (5.5)	1.10 (0.93–1.30)
Household income quartile (by zip code)			
First quartile (bottom)	648,724 (29.9)	5287 (35.7)	1.53 (1.37–1.71)
Second quartile	546,750 (25.2)	3880 (26.2)	1.33 (1.18–1.49)
Third quartile	538,072 (24.8)	3317 (22.4)	1.16 (1.03–1.31)
Fourth quartile (top)	436,098 (20.1)	2325 (15.7)	Ref
Insurance Type			
Medicare	17,357 (0.8)	652 (4.4)	7.47 (6.21–8.98)
Medicaid	902,572 (41.6)	7716 (52.1)	1.70 (1.58–1.84)
Private	1,154,251 (53.2)	5791 (39.1)	Ref
Self‐pay	41,223 (1.9)	207 (1.4)	1.06 (0.78–1.44)
No charge	too few	too few	1.90 (0.61–5.92)
Other	54,241 (2.5)	443 (3.0)	1.58 (1.26–1.96)
Hospital location/ teaching status			
Rural	154,045 (7.1)	874 (5.9)	Ref
Urban non‐teaching	327,616 (15.1)	1,851 (12.5)	0.98 (0.82–1.17)
Urban teaching	1,687,983 (77.8)	12,085 (81.6)	1.25 (1.07–1.45)
Hospital type and ownership			
Government	282,054 (13.0)	2,296 (15.5)	Ref
Private, non‐profit	1,635,912 (75.4)	10,945 (73.9)	0.82 (0.74–0.91)
Private profit	251,679 (11.6)	1,570 (10.6)	0.77 (0.66–0.89)
Hospital bed size			
Small	384,027 (17.7)	2,340 (15.8)	Ref
Medium	611,840 (28.2)	3,954 (26.7)	1.06 (0.95–1.19)
Large	1,173,777 (54.1)	8,516 (57.5)	1.19 (1.08–1.32)
Year			
2017	368,839 (17.0)	2,429 (16.4)	Ref
2018	407,893 (18.8)	2,873 (19.4)	1.07 (0.95–1.21)
2019	438,268 (20.2)	2,918 (19.7)	1.01 (0.89–1.14)
2020	457,795 (21.1)	3,140 (21.2)	1.05 (0.93–1.18)
2021	496,848 (22.9)	3,451 (23.3)	1.05 (0.94–1.18)

*Note*: Data presented as *N* (%) or mean ± standard error.

Abbreviations: BMI, body mass index; CI, confidence interval; OR, odds ratio.

**TABLE 4:** Outcomes among those with hypertensive disorders of pregnancy only.

	No history of seizure disorder (*N* = 2,169,644)	History of seizure disorder (*N* = 14,810)	OR /Coef	95% CI	aOR/Coef	95% CI
Gestational age at delivery	37.3 ± 0.0	36.5 ± 0.1	−0.71	−0.80 to −0.62	−0.55	−0.64 to −0.46
Preterm delivery	503,357 (23.2)	4,769 (32.2)	1.39	1.32–1.46	1.41	1.30–1.53
Cesarean section delivery	43.0 (932,947)	51.3 (7,598)	1.19	1.15–1.24	1.38	1.28–1.49
Length of stay (days)	3.6 ± 0.0	4.2 ± 0.1	0.57	0.46 to 0.68	0.44	0.33 to 0.55
Hemorrhage	164,893 (7.6)	1,288 (8.7)	1.15	1.02–1.29	1.16	1.02–1.32
Suspected FGR	134,518 (6.2)	1,111 (7.5)	1.20	1.06–1.37	1.12	0.97–1.28
IUFD	13,018 (0.6)	118 (0.8)	1.54	1.04–2.28	1.28	0.86–1.91
Abruption	34,714 (1.6)	281 (1.9)	1.18	0.91–1.52	1.09	0.84–1.42
HELLP	45,563 (2.1)	474 (3.2)	1.54	1.25–1.89	1.65	1.34–2.02
Eclampsia	10,848 (0.5)	1,451 (9.8)	22.73	19.99–25.85	20.90	18.31–23.86
SMM	119,330 (5.5)	2,607 (17.6)	3.22	2.97–3.48	3.37	3.06–3.72
SMM excluding transfusion	75,938 (3.5)	2,207 (14.9)	4.29	3.93–4.68	4.36	3.93–4.84
SMM excluding eclampsia	110,652 (5.1)	1,762 (11.9)	2.32	2.10–2.56	2.27	2.02–2.54
Death	11/100,000	34/100,000	3.12	0.43–22.59	2.53	0.35–18.60

Abbreviations: CI, confidence interval; FGR, fetal growth restriction; IUFD, intrauterine fetal demise; HELLP, hemolysis, elevated liver enzymes, low platelets; OR, odds ratio; SMM, severe maternal morbidity.

**FIGURE 2:**
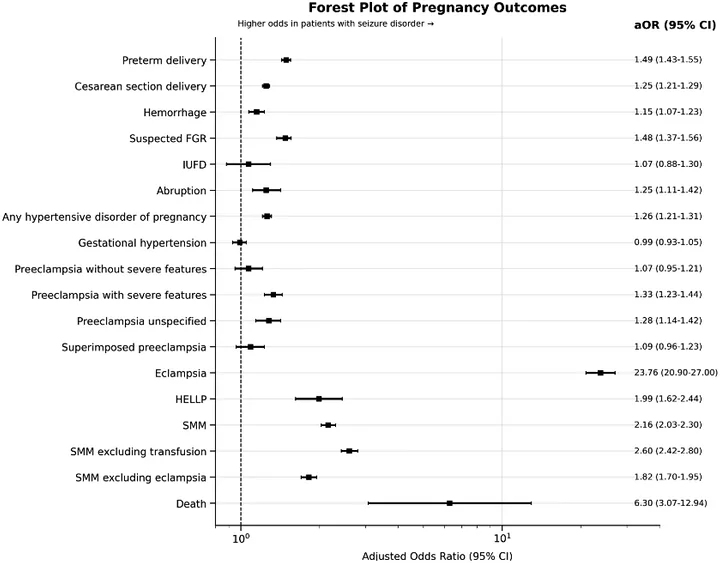
Forest plot of outcomes among those with HDP only. HDP, hypertensive disorders of pregnancy.

E‐value analyses (Supplement 3 in the Supporting Information) indicated that the associations with SMM were relatively robust to unmeasured confounding. An unmeasured confounder would need to be associated with both seizure disorder and SMM by risk ratios of at least 6.20 each to explain away the overall SMM association. The corresponding E‐values were 8.19 for SMM excluding transfusion and 3.97 for SMM excluding eclampsia. In contrast, the associations with preterm delivery, cesarean delivery, and hemorrhage had smaller E‐values, suggesting greater sensitivity to unmeasured confounding. E‐values for outcomes with CIs that included the null were 1.00 at the confidence‐limit level and therefore provided no additional evidence of robustness (i.e., IUFD, placental abruption, and maternal death).

## 4 DISCUSSION

Overall, patients with an ICD‐10 code for seizure disorder had higher odds of almost every adverse obstetric outcome analyzed in this study. As hypothesized, patients with a history of seizure disorder showed increased odds for a composite of SMM, both including and excluding transfusions and eclampsia. Patients with a history of seizure disorder also had an earlier gestational age at delivery, higher rates of cesarean section, placental abruption, postpartum hemorrhage, fetal growth restriction, preterm delivery, preeclampsia with severe features, eclampsia, HELLP syndrome, and maternal death, but no change in rate of stillbirth.

We observed a striking sixfold increased odds of maternal death among patients with seizure disorder. This is lower than a study of NIS 2007–2011 data that found a 10‐fold increased risk of maternal death [7]. Similarly, using the CDC's 21 SMM indicators [8], we found that patients with a history of seizure disorders had more than twice the odds of experiencing a composite SMM outcome compared with patients without a history of seizure disorder. These findings are similar to a large study conducted in Denmark [11]. The lower rates of maternal mortality and SMM in our study compared to historical data may reflect advances in awareness and clinical practice, leading to more individualized monitoring and management of pregnant patients during the antepartum period. For example, in 2009, the AAN and the American Epilepsy Society (AES) published an evidence‐based Practice Parameter that updated guidance on the management of patients with seizure disorder during pregnancy, including evidence that the absence of seizures during the nine months preceding conception is associated with a high likelihood of remaining seizure‐free throughout pregnancy [6]. A 2012 study found that patients who experienced a seizure in the month preceding pregnancy were 15 times more likely to experience a seizure during pregnancy [18]. Conversely, more than 80% of patients who remained seizure‐free during the year preceding pregnancy remained seizure‐free throughout pregnancy [19]. Appropriate preconception counseling and management may facilitate optimization of seizure control prior to conception. The higher rate of seizures and anti‐seizure medication burden observed among unintended pregnancies may reflect a missed opportunity for preconception optimization of seizure control, which is more feasible among patients with planned pregnancies [20].

Multiple studies have demonstrated the safety profiles of the anti‐seizure medications most commonly used during pregnancy [21, 22]. Consequently, the use of anti‐seizure medications during pregnancy is now standard practice, as the risks associated with uncontrolled seizures generally outweigh the potential risks associated with pharmacologic therapy. In 2015, a paper published in the *Expert Review of Neurotherapeutics* identified the need for serial monitoring of anti‐seizure medication levels to ensure therapeutic doses in every trimester, reducing morbidity from inadequate seizure control [23]. The Maternal Outcomes and Neurodevelopmental Effects of Antiepileptic Drugs (MONEAD) study, published in 2022, evaluated changes in anti‐seizure medication concentrations throughout pregnancy. Significant declines in serum concentrations were observed for lamotrigine, levetiracetam, lacosamide, oxcarbazepine, and zonisamide as pregnancy progressed, further underscoring the importance of serial therapeutic drug monitoring and dose adjustment during pregnancy [24]. These updated guidelines likely contributed to the decreased risk of maternal mortality and SMM seen from 2007–2011 to our findings in 2017–2021.

The increased risk for preeclampsia with severe features, HELLP syndrome, and eclampsia in patients with seizure disorder confirm findings established in prior studies [7, 10, 11, 25]. While the mechanism is unclear, studies have found that endocrine changes, particularly fluctuations in female sex steroid hormones and their neuroactive metabolites, modulate brain excitability through both short‐acting neuronal membrane‐mediated effects and long‐acting genomic effects [26]. It is unclear why all types of HDP are not increased in patients with seizure disorder diagnoses. This may be due to the great deal of heterogeneity of pathogenesis within the subtypes of HDP, and certain subtypes may have more similar pathogenesis to seizures than others [27].

Although our study demonstrated a 23‐fold increased risk of eclampsia, it is challenging to determine whether seizures in this population were attributable to eclampsia or to an underlying seizure disorder. In clinical practice, distinguishing between eclamptic seizures and seizures related to a pre‐existing seizure disorder can be difficult. To further examine this issue, we stratified our analysis by the presence of HDP. Among patients with HDP and a seizure disorder, the rate of eclampsia was still significantly higher than among those with HDP but without a seizure disorder; however, the overall difference between eclampsia rates was slightly less striking when analyzing only those with HDP, demonstrating that some epileptic seizures are likely classified as eclampsia, but that they are at a very high risk of seizure activity regardless of meeting other criteria for HDP. Although the rate of SMM excluding eclampsia remained significantly higher among patients with a history of seizure disorder, misclassification of epileptic seizures as eclampsia may have introduced substantial bias and represents an additional limitation of our study.

Most of the recommendations from national societies including ACOG, Society for Maternal‐Fetal Medicine (SMFM), and the AAN center on the approach to anti‐seizure medication choice in pregnancy to decrease fetal anomalies and congenital malformations. In contrast, there is insufficient research and subsequent recommendations about maternal outcomes and their mechanisms. For example, the increased risk of maternal death is hypothesized to occur due to complications of seizures (such as aspiration or sudden unexpected death in seizure disorder) [7], but no definitive mechanism has been established. A publication reviewing the 2011 report of the United Kingdom Confidential Enquiries into Maternal Deaths identified a potential association between anti‐seizure medication use and maternal mortality, noting that 64% of patients who died were receiving lamotrigine therapy, although lamotrigine can be used for other non‐seizure related disorders [28]. Future research can investigate these mechanisms as this understanding would help to further customize antepartum care for these patients and continue to decrease rates of mortality and SMM.

Our large sample size allows for more generalizable results and reduces the impact of individual variation. The wide range of primary and secondary outcomes analyzed helps gain a broad understanding of how a seizure disorder in pregnancy can impact maternal health. Our study was limited by the use of ICD‐10 codes and thus there is no information on the severity of seizure disorder or the use of anti‐seizure medications in pregnancy, limiting the patient‐specific associations of our findings. For example, it is possible that seizures not related to seizure disorder (substance‐related, hypoglycemia, etc.) were miscoded as epileptic seizures. Functional seizures, otherwise known as psychogenic nonepileptic seizures (PNES), involve episodes that may resemble seizure‐like presentations. These episodes may also have been misclassified as epileptic seizures, falsely skewing the data. Additionally, our study may have overlooked well‐controlled seizure disorder that was not reflected in obstetric records as patients were not on anti‐seizure medications or had not had a seizure in many years. It is also important to include that ICD‐10 codes do not have ideal validity for SMM, as coding is built for billing and not research, so the use of these codes is a limitation of our study [29]. However, use of ICD‐10 codes is beneficial in order to study rare outcomes such as SMM.

Use of a large dataset introduces other limitations, such as the inability to know whether the incidence of seizure disorder has truly increased or if coding has just become more accurate during the period of time investigated in this study due to increased awareness amongst clinicians or more complete documentation (ascertainment bias). As a result, observed increases in seizure disorder diagnoses over time may reflect evolving coding practices and improved recognition rather than a true increase in disease burden. Our findings may also be subject to selection bias, as inclusion in the database was contingent upon interaction with the healthcare system. Patients with more severe or symptomatic seizure disorders are more likely to seek medical care and be included in the database, which may have resulted in an overestimation of the association between seizure disorder and adverse pregnancy outcomes. Although residual confounding cannot be excluded, the E‐values for SMM suggest that a strong unmeasured confounder would be required to explain away the observed associations we identified.

Of note, study numbers varied in our sensitivity analysis because different eligibility criteria were applied (HDP only) and observations were reweighted to reflect the characteristics of the specific subgroup. We suspect that the effective sample size and weighted distribution differed from those of the primary analysis, despite being derived from the same underlying population, leading to slightly different *N* values.

Our study is likely limited by differential reporting or documenting in patients with a seizure disorder versus those without a seizure disorder. Because patients with a seizure disorder may be more medically complex and require closer monitoring, it is possible that their comorbidities and baseline chronic conditions were more commonly reported than those without seizure disorder.

Despite the large sample size, this study lacked detailed clinical information regarding seizure disorder characteristics due to the reliance on ICD‐10 codes through the NIS. Future research should examine how factors such as seizure control, seizure type, seizure frequency before and during pregnancy, and other measures of disease severity influence maternal and neonatal outcomes. Additionally, because our analysis was limited to the delivery hospitalization, antepartum complications requiring hospitalization would not have been captured in our study.

The findings of our study have the potential to inform clinical practice by reinforcing the need for individualized prenatal care in pregnancies complicated by maternal seizure disorder. Improved recognition of the associated maternal and perinatal risks may guide preconception counseling, risk stratification, and shared decision‐making throughout pregnancy. These findings may also influence the frequency of prenatal surveillance, the use of antenatal testing, intrapartum planning, multidisciplinary support, and postpartum follow‐up.

## 5 CONCLUSION

Seizure disorder impacts 1.5 million people of reproductive age and 0.3–0.7% [30] of pregnancies. Past and current research have shown increased risk of adverse maternal and neonatal outcomes among those with a seizure disorder in pregnancy. Over the past 10 years, we have seen some improvement in maternal mortality rates and SMM. To our knowledge, this is the largest study conducted in a contemporary cohort in the United States examining maternal outcomes among those with seizure disorder in pregnancy. While our study provided updated estimates on the rates of maternal outcomes in those with seizure disorders, future research should investigate the mechanisms driving these outcomes to help customize care and mitigate complications.

## FUNDING INFORMATION

The authors received no specific funding for this work.

## CONFLICT OF INTEREST STATEMENT

The authors declare no conflicts of interest.

## ETHICS STATEMENT

Institutional review board approval was waived as this is a nationally, publicly available dataset.

## Supporting information



Supporting Information

Supporting Information

Supporting Information

## Data Availability

Data are publicly available through the AHRQ‐HCUP data warehouse.
