# Supplementary material for: Severe maternal morbidity in pregnancies complicated by maternal seizure disorder
Source: Pregnancy (Hoboken). 2026 Sep 29;2(6):e70466. doi: 10.1002/pmf2.70466 (PMC13620848; doi:10.1002/pmf2.70466)
Supplement: Supplementary file 1 [file PMF2-2-e70466-s001.pdf]

Supplementary Table 3. E-values for sensitivity to unmeasured confounding

Outcome	Adjusted association (95% CI)	Point-estimate E-value	CI-limit E-value
Gestational age at delivery	β = −0.55 (−0.64 to −0.46)	Not calculated	Not calculated
Preterm delivery	OR = 1.41 (1.30 to 1.53)	1.66	1.54
Cesarean delivery	OR = 1.38 (1.28 to 1.49)	1.63	1.52
Hospital length of stay	β = 0.44 (0.33 to 0.55)	Not calculated	Not calculated
Hemorrhage	OR = 1.16 (1.02 to 1.32)	1.59	1.16
Suspected fetal growth restriction	OR = 1.12 (0.97 to 1.28)	1.49	1.00
Intrauterine fetal death	OR = 1.28 (0.86 to 1.91)	1.88	1.00
Placental abruption	OR = 1.09 (0.84 to 1.42)	1.40	1.00
HELLP syndrome	OR = 1.65 (1.34 to 2.02)	2.69	2.01
Eclampsia	OR = 20.90 (18.31 to 23.86)	41.29	36.11
Severe maternal morbidity	OR = 3.37 (3.06 to 3.72)	6.20	5.57
Severe maternal morbidity excluding transfusion	OR = 4.36 (3.93 to 4.84)	8.19	7.32
Severe maternal morbidity excluding eclampsia	OR = 2.27 (2.02 to 2.54)	3.97	3.46
Maternal death	OR = 2.53 (0.35 to 18.60)	4.50	1.00

a. The E-value is the minimum strength of association, on the risk-ratio scale, that an unmeasured confounder would need to have with both the exposure and outcome, conditional on measured covariates, to fully explain away the observed association.

b. The CI-limit E-value corresponds to the 95% CI bound closest to the null. It is 1.00 when the 95% CI includes the null.

c. For the common outcomes of preterm delivery and cesarean delivery, odds ratios were approximately converted to the risk-ratio scale using the square-root transformation before E-values were calculated. For the remaining binary outcomes, odds ratios were treated as approximations of risk ratios. This approach should be verified against weighted outcome prevalences before publication.

d. E-values were not calculated for gestational age at delivery or hospital length of stay because the reported estimates are unstandardized coefficients for continuous outcomes. Calculation requires an outcome standard deviation—preferably the model residual standard deviation—to convert each coefficient to a standardized effect and then to the risk-ratio scale; this information cannot be recovered from the coefficient and 95% CI alone.

e. E-values assess sensitivity to unmeasured confounding only; they do not address selection bias, exposure or outcome misclassification, or model misspecification.

Abbreviations: CI, confidence interval; HELLP, hemolysis, elevated liver enzymes, and low platelet count; OR, odds ratio.
