# Supplementary material for: Severe maternal morbidity in pregnancies complicated by maternal seizure disorder
Source: Pregnancy (Hoboken). 2026 Sep 29;2(6):e70466. doi: 10.1002/pmf2.70466 (PMC13620848; doi:10.1002/pmf2.70466)
Supplement: Supplementary file 2 [file PMF2-2-e70466-s002.pdf]

Supplement 1: ICD-10 Codes

	ICD-10 Diagnosis or Procedure Codes
**Obesity (body mass index 30.0 kg/m^2 or greater)**	O99.210-O99.215 E66.0-E66.2 E66.8-E66.9 Z68.3 Z68.4 Z68.30-Z68.99
**Tobacco use**	F17.200 F17.203-F17.209 F17.210 F17.213-F17.219 F17.220 F17.223-F17.229 F17.290 F17.293-F17.299 O99.330-O99.335
**Diabetes mellitus**	O24.0-O24.33 O24.80-O24.93 E10.00-E10.90 E11.00-E11.90 E13.00-E13.90
**Chronic hypertension**	O10.0-O10.93 I10 I10.0 I11.0 I11.9 I12.0 I12.9 I13 I13.0-I13.9 I15 I15.0-I15.9 O16.1 I16 I16.0-I16.9 I1AI 1A.0
**Asthma**	J45.2-J45.998 O99.5 O99.519
**Anemia**	O90.81 O99.0-O99.019 D50.9 D64.9-D64.901
**Mood disorder**	F33.0-F33.9 F32.0-F32.9 F32.A F30.0-F30.9 F31.0-F31.9 34.0-F34.9 F39.0-F39.9 O99.340-O99.343 F41.0-F41.9
**History of cesarean**	O34.2-O34.22 O66.41
**Gestational hypertension**	O13.0-O13.9
**Preeclampsia without severe features**	O14.00-O14.05
**Preeclampsia with severe features**	O14.10-O14.19
**Superimposed preeclampsia**	O11.00-O11.90
**HELLP syndrome/eclampsia**	O14.20-O14.29
**Eclampsia**	O15.00-O15.90
**Gestational age at delivery**	Z3A.xx
**Cesarean delivery**	10D00Z0-10D00Z2 10A00ZZ 10A03ZZ 10Z04ZZ
**SMM**	
**Acute MI**	I21.01-I21.9 121.A1 I21.A9 I22.0-I22.9
**Aneurysm**	I71.00–I71.03 I71.1-I71.9 I79.0
**Acute renal failure**	N17.0-N17.9 N19 O90.4 N99.0
**Acute respiratory distress**	J80 J95.1 J95.2 J95.3 J95.821 J95.822 J96.00 J96.01 J96.02 J96.20 J96.21 J96.22 R09.2
**Amniotic fluid embolism**	O88.110-O88.119 O88.12 O88.13
**Cardiac Arrest / Ventricular Fibrillation**	I46.2 I46.8 I46.9 I49.01 I49.02
**Conversion of Cardiac Rhythm**	5A12012, 5A2204Z
**DIC**	D65, D68.8, D68.9, O45.002, O45.003, O45.009, O45.012, O45.013, O45.019, O45.022, O45.023, O45.029, O45.092, O45.093, O45.099, O46.002, O46.003, O46.009, O46.012, O46.013, O46.019, O46.022, O46.023, O46.029, O46.092, O46.093, O46.099, O67.0, O72.3
**Blood transfusion**	30233H1 30240H1 30243H1 30233K1 30233L1 30233M1 30240K1 30240L1 30240M1 30243K1 30243L1 30243M1 30233N1 30233P1 30240N1 30240P1 30243N1 30243P1 30233N0 30233P0

	30240N0 30240P0 30243N0 30243P0 30233R1 30240R1 30243R1 30233T1 30240T1 30243R1
**Eclampsia**	O15.00-O15.90
**Cardiac failure, cardiac arrest**	I97.120-I97.131 I97.710 I97.711
**CVAT**	I60.0-I60.9 I61.1-I61.9 I62.0-I62.9 I63.0-I63.9 I65.0-I65.9 I66.0-I66.9 I67.0-I67.9 I68.0-I68.8 G45.0-G45.9 G46.0-G46.8 O22.51-O22.53 I97.810-I97.821 O87.3
**Heart failure, pulmonary edema**	J81.0 I50.1 I50.20 I50.21 I50.23 I50.30 I50.31 I50.33 I50.40 I50.41 I50.43 I50.9 J68.1
**Anesthesia complications**	O29.0-O29.93 O29.5X-O29.5X9 O29.3X-O29.3X9 O74.0-O74.3 O89.01 O89.09 O89.1 O89.2 T41.0-T41.5 T88.2-T88.59 O29.60-O29.63 O29.011-O29.293 O29.8X1-O29.8X3 O29.91-O29.93 O74.0-O74.3 O74.7-O74.9 O89.09 O89.8 O89.9 O29.5X1-O29.5X9 O89.4 T88.59 O74.5 O29.41-O29.43 O89.5 O75.6 O29.3X1-O29.3X3 O74.4 O74.5 O74.6
**Sepsis**	A02.1 A40.0 A40.1 A40.3 A40.8 A40.9 A41.0-A41.9 A32.7 A42.7 T81.44 T81.44XA T81.44XD T81.44XS O75.3 R78.81 O85 O86.04 T81.4 R65.2 R65.20 R65.21 B37.7 B00.7
**Shock**	O75.1 R57.0 R57.1 R57.8 R57.9 R65.21 T78.2XXA T88.2XXA T88.6XXA T81.10XA T81.11XA T81.19XA
**Sickle cell crisis**	D57.00 D57.01 D57.02 D57.211 D57.212 D57.219 D57.411 D57.412 D57.419 D57.811 D57.812 D57.819
**Embolism (air, thromboembolic, arterial, venous)**	I26.01 I26.02 I26.09 I26.90 I26.92 I26.99 I74.0-I74.9 I81 I82.0 I82.210 I82.220 I82.290 I82.401-I82.499 I82.4Y0-I82.4Y9 I82.4Z0-I82.4Z9 I82.600-I82.609 I82.620-I82.629 I82.A10-I82.A19 I82.B10-I82.B19 I82.C10-I82.C19 I82.890 I82.90 O04.7 O22.32-O22.33 O88.011-O88.019 O88.02 O88.03 O88.211-O88.219 O88.22 O88.23 O88.311-O88.319 O88.32 O88.33 O88.81 O88.82 O88.83
**Hysterectomy**	0UT90ZZ 0UT94ZZ 0UT97ZZ 0UT98ZZ 0UT9FZZ 0UB9 0UT9 0UT90ZL 0UT97ZL 0UT98ZL 0UT9FZL
**Tracheostomy**	0B110Z4 0B110F4 0B113Z4 0B113F4 0B114Z4 0B114F4
**Ventilator**	5A1935Z 5A1945Z 5A1955Z

MSDRG codes used to identify delivery encounters:

- MS-DRG 768 Vaginal Delivery with O.R. procedure except Sterilization &/or D&C
- MS-DRG 783 Cesarean Section with Sterilization with MCC
- MS-DRG 784 Cesarean Section with Sterilization with CC
- MS-DRG 785 Cesarean Section with Sterilization without CC/MCC
- MS-DRG 786 Cesarean Section without Sterilization with MCC
- MS-DRG 787 Cesarean Section without Sterilization with CC
- MS-DRG 788 Cesarean Section without Sterilization without CC/MCC
- MS-DRG 796 Vaginal Delivery with Sterilization/D&C with MCC

MS-DRG 797 Vaginal Delivery with Sterilization/D&C with CC
MS-DRG 798 Vaginal Delivery with Sterilization/D&C without CC/MCC
MS-DRG 805 Vaginal Delivery without Sterilization/D&C with MCC
MS-DRG 806 Vaginal Delivery without Sterilization/D&C with CC
MS-DRG 807 Vaginal Delivery without Sterilization/D&C without CC/MCC

The following are data elements within the NIS: age, race/ethnicity as reported by the hospital, patient location by population, household income quartile, insurance type, hospital location and teaching status, hospital type and ownership, hospital bed size, and year of admission.
